# ﻿Discovery of *Dichocrocisfrenatalis* Lederer, 1863 (Lepidoptera, Crambidae, Spilomelinae) in mangrove environments of the Ryukyu Islands, Japan, and tribal placement of the genus

**DOI:** 10.3897/zookeys.1243.155924

**Published:** 2025-06-25

**Authors:** Yuki Matsui, Nakatada Wachi, Yutaka Yoshiyasu

**Affiliations:** 1 Entomological Laboratory, Faculty of Agriculture, Kyushu University, 744 Motooka, Nishi-ku, Fukuoka 819-0395, Japan Kyushu University Fukuoka Japan; 2 Iriomote Station, Tropical Biosphere Research Center, University of the Ryukyus, 870 Uehara, Taketomi, Yaeyama, Okinawa 907-1541, Japan University of the Ryukyus Okinawa Japan; 3 Laboratory of Applied Entomology, Graduate School of Life and Environmental Science, Kyoto Prefectural University, Shimogamo, Kyoto 606-8522, Japan Kyoto Prefectural University Kyoto Japan

**Keywords:** Adults aggregation, hair-pencils, polyphyletic genus, Steniini, type species

## Abstract

*Dichocrocisfrenatalis* Lederer, 1863, the little-known type species of the polyphyletic genus *Dichocrocis*, was discovered in the Ryukyu Islands, Japan. We provide a redescription of the adult morphology, including the male genitalia with highly developed hair-pencil structures, as well as female genitalia. The phylogenetic placement of this genus, and therefore its type species, within Spilomelinae has been uncertain, but our molecular phylogenetic analysis and morphological evaluation support the placement of the genus *Dichocrocis* in the tribe Steniini. Additionally, as a novel ecological observation, we report that the Japanese population of this species is abundant in mangrove environments, where the adults frequently aggregate on the underside of leaves and are preyed upon by a mangrove-associated robber fly.

## ﻿Introduction

The genus *Dichocrocis* Lederer, 1863 was established by monotypy for *D.frenatalis* Lederer, 1863 from the Nicobar Islands, India. Hampson (1896) synonymized the type species with *Botyspandamalis* Walker, 1859 and expanded the genus definition based on unreliable external morphological evidence such as the shape of palpi and legs, and wing venation. Although Hampson’s classification was heterogeneous, as it included species belonging to *Conogethes*, *Gadessa*, *Orthospila*, and *Rehimena*, each of which is now recognized as valid genera, it was nevertheless followed by many subsequent authors (e.g., Pajni and Rose 1977; Mandal and Bhattacharya 1979). Among these genera, the separation of *Conogethes* from *Dichocrocis* became widely accepted after the 1980s (e.g., Inoue 1982; Inoue and Yamanaka 2006), but no formal explanation was provided for this treatment. Recently, Mally et al. (2019) transferred *D.pandamalis* to *Conogethes* based on morphological and molecular evidence. Currently, *D.frenatalis*, the genus type species, is regarded as a valid species, and the genus *Dichocrocis* comprises 53 described species mainly from the Paleotropics (Nuss et al. 2025). However, the original description of the type species provides only a brief account of its external morphology, and no detailed morphological study has been conducted on this species to date. As a result, the taxonomic definition and diagnostic characters of the genus remain ambiguous. Although Mally et al. (2019) examined the male genitalia of *D.frenatalis* (from Sumatra, Indonesia) and suggested its tribal placement in Steniini, they did not formally assign the genus to that tribe.

No *Dichocrocis* species had previously been recorded in Japan. Recently, however, we discovered *D.frenatalis* on Okinawa, Ishigaki, and Iriomote Islands, in the Ryukyu Islands of southwestern Japan. In this study, we redescribe *D.frenatalis* and assign *Dichocrocis* to a spilomeline tribe based on morphological investigations and molecular phylogenetic analysis. We also report for the first time the occurrence of this species in a mangrove environment on Iriomote Island, where the adults frequently aggregate on the underside of leaves and are preyed upon by a mangrove-associated robber fly.

## ﻿Materials and methods

Most *Dichocrocisfrenatalis* specimens were collected by daytime search in a mangrove environment on Iriomote Island (Funaura), with some additional specimens obtained through light trapping at various localities on Okinawa, Ishigaki, and Iriomote Islands. The specimens were deposited in the Entomological Laboratory of Kyushu University, Japan (**ELKU**); the Laboratory of Environmental Zoology and Entomology, Osaka Metropolitan University, Japan (**OMU**); the Ryukyu University Museum (Fujukan), Japan (**RUMF**); or the Iriomote Station, Tropical Biosphere Research Center, University of the Ryukyus, Japan (**TBRC**). The specimens preserved at the National Museum of Nature and Science, Japan (**NSMT**) were also examined. Methods for morphological observation and genitalia dissection are described in Matsui et al. (2024). Morphological terminology follows Maes (1985) for tympanal organs, Downey and Allyn (1975) for hair-pencil scales, and Mally et al. (2019) for all other structures. Forewing lengths of males and females were compared using Welch’s two-sample *t*-test in R v. 4.4.2 implemented in WebR (https://github.com/r-wasm/webr). Mean and standard deviation (SD) of the forewing lengths were also calculated for each sex.

One specimen of *D.frenatalis* (♂, Funaura, Iriomote Island, 14 July 2023, NW leg.) was used for DNA analysis. The methods for DNA extraction, PCR reaction, and sequencing follow Matsui et al. (2022, 2024). Briefly, the entire COI gene and fragments of genes RpS5, CAD, and EF1α were sequenced using primer sets described in Matsui et al. (2022). The obtained sequences were aligned and manually corrected using MEGA v. 7.0 (Kumar et al. 2016) with the dataset of the same regions by Matsui et al. (2022). A maximum-likelihood tree was constructed using IQ-TREE v. 2.3.1 (Minh et al. 2020). The concatenated dataset was partitioned by gene and codon position, and the best-fitting nucleotide substitution models were selected using ModelFinder implemented in IQ-TREE based on the Bayesian information criterion (BIC). Branch supports were calculated using Ultrafast Bootstrap (UFBT) and Shimodaira-Hasegawa-like approximate likelihood ratio tests (SH-aLRT) implemented in IQ-TREE each with 1,000 replicates. The obtained sequences were deposited in DDBJ (https://www.ddbj.nig.ac.jp/) (accession numbers: COI: LC867066, RpS5: LC867065, CAD: LC867063, and EF1α: LC867064).

English editing was supported by OpenAI’s ChatGPT (GPT-4) under the supervision of the authors.

## ﻿Results

### ﻿Taxonomy

#### ﻿ Dichocrocis frenatalis

Taxon classificationAnimaliaLepidopteraCrambidae

Lederer, 1863

6ED531DE-BF09-56B8-952C-7E5BB9386CB9

**Figure 1.**
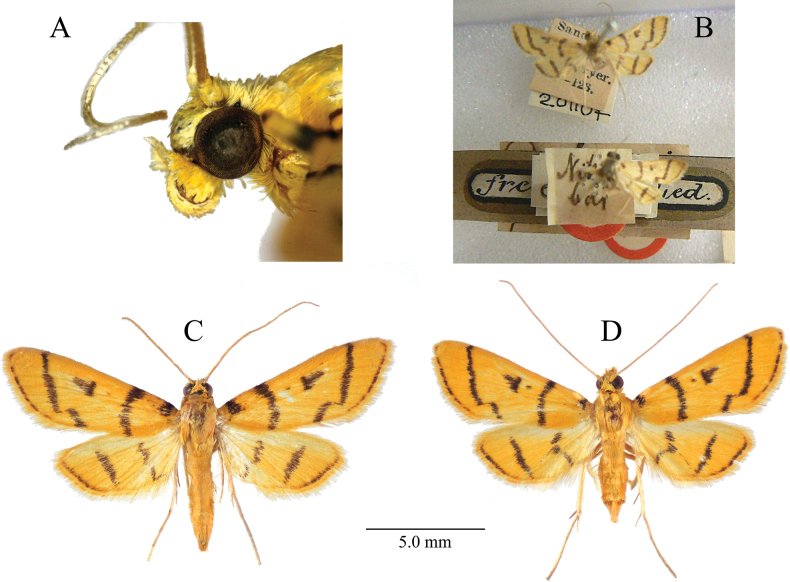
*Dichocrocisfrenatalis* adults. **A.** Head; **B.** The holotype (bottom) preserved in NHMUK (photo by Richard Mally); **C.** Male; **D.** Female.

**Figure 2.**
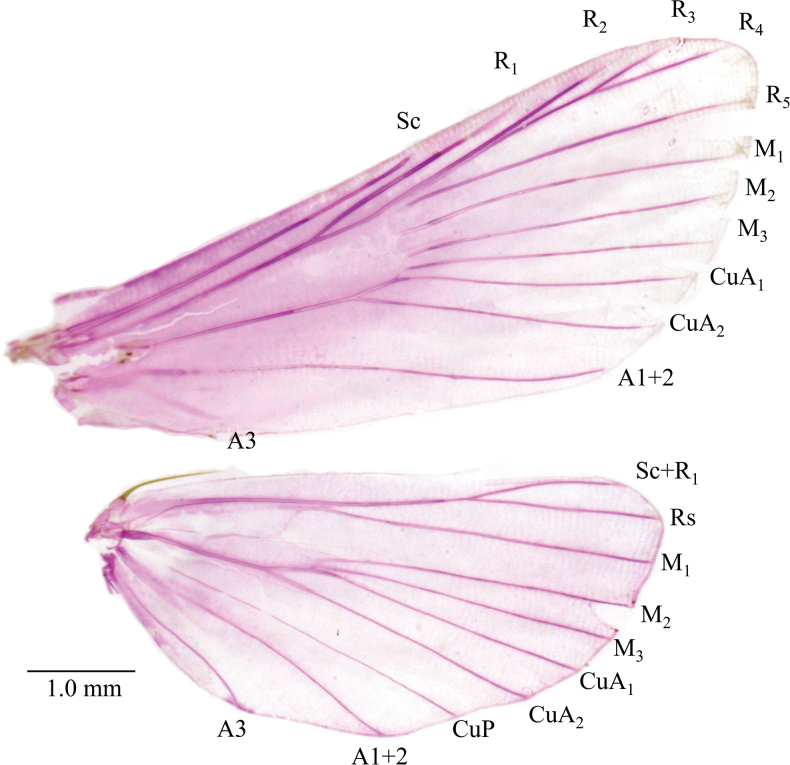
*Dichocrocisfrenatalis* female wing venation.

**Figure 3.**
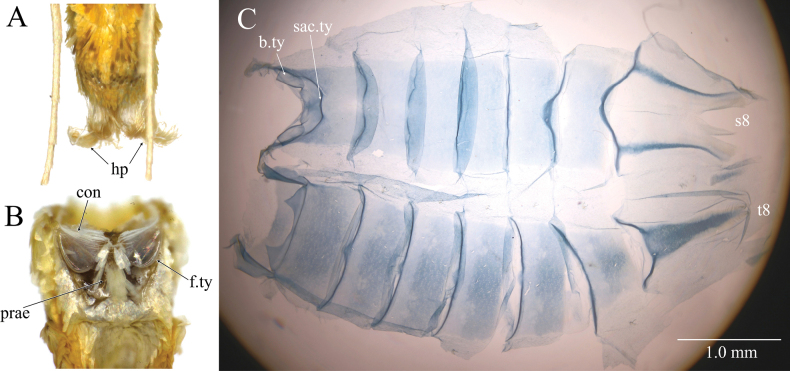
*Dichocrocisfrenatalis* abdominal structures. **A.** Male abdominal tip with visible hair-pencils (indicated by arrows); **B.** Tympanal organs; **C.** Male abdominal cuticle (slide no. YM878). b.ty: bulla tympani, con: conjunctivum, f.ty: fornix tympani, hp: hair-pencils, prae: praecinctorium, sac.ty: saccus tympani, s8: sternite 8, t8: tergite 8.

**Figure 4.**
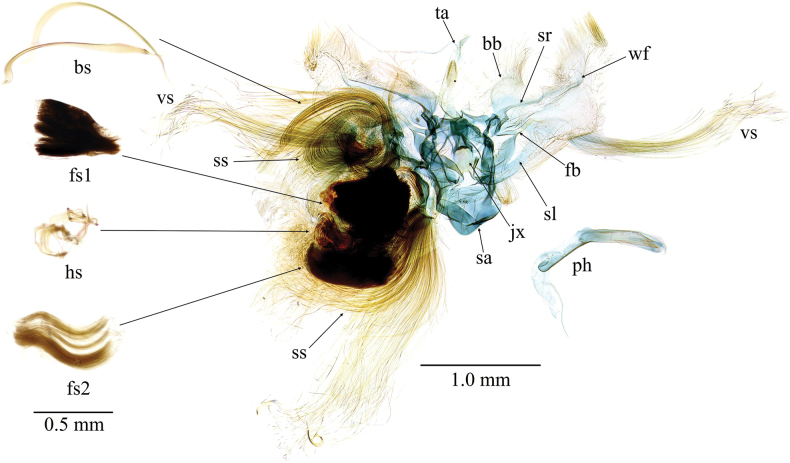
*Dichocrocisfrenatalis* male genitalia (slide no. YM878). bb: basal bulge of valva costa, bs: broad spatulate scale, fb: fibula, fs1: filiform scale 1, fs2: filiform scale 2, hs: hook-shaped scale, jx: juxta, ph: phallus, sa: saccus, sl: sacculus, sr: sclerotized ridge of valva costa; ss: slender spatulate scale, ta: tuba analis, vs: valva setae, wf: wavy furrow.

**Figure 5.**
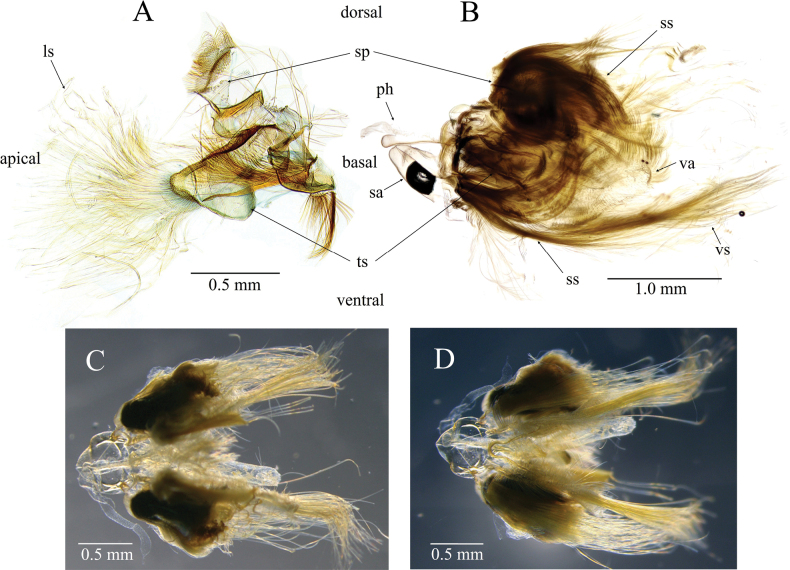
*Dichocrocisfrenatalis* male genitalia. **A.** Parateguminal sclerites (most scales removed, slide no. YM1276); **B.** Whole genitalia, unmounted, left lateral view (internal hair-pencil scales removed); **C.** Whole genitalia, unmounted, dorsal view; **D.** Ditto, dorsal view; **D.** Ditto, ventral view. ls: lamellate scale 1, ph: phallus, sa: saccus, sp: saddle-shaped pad, ts: triangular sclerite, ss: slender spatulate scale, vs: valva setae.

**Figure 6.**
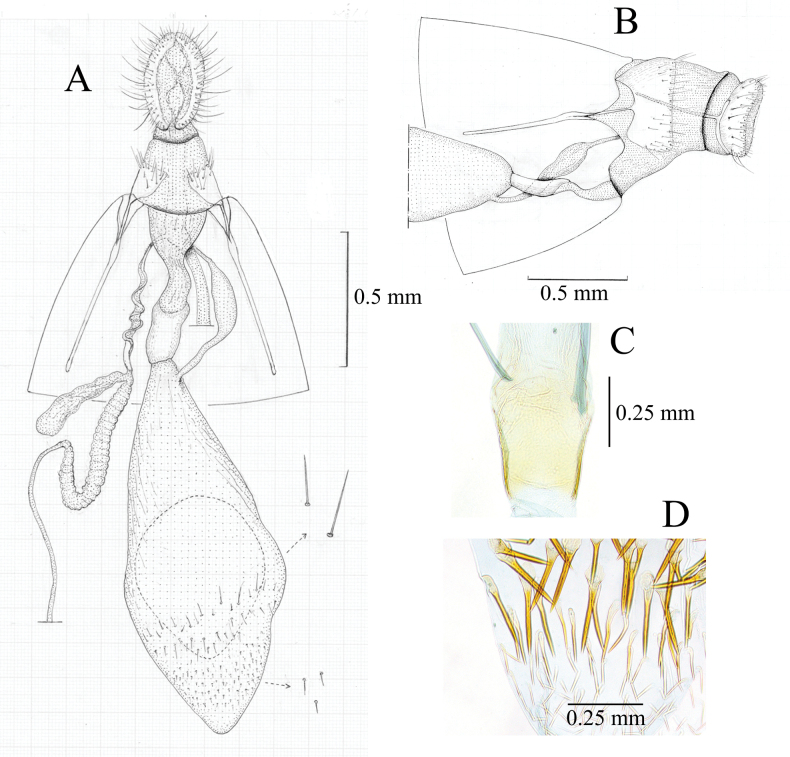
*Dichocrocisfrenatalis* female genitalia. **A.** Whole genitalia, ventral view, spermatophore in the corpus bursae indicated by dotted circle (illustration by YY); **B.** 8^th^ to 10^th^ abdominal segments, lateral view (spermathecal gland excluded); **C.** Sclerotization of ductus bursae, magnified view (slide no. YM877); **D.** Spines on corpus bursae, magnified view (slide no. YM877).

**Figure 7.**
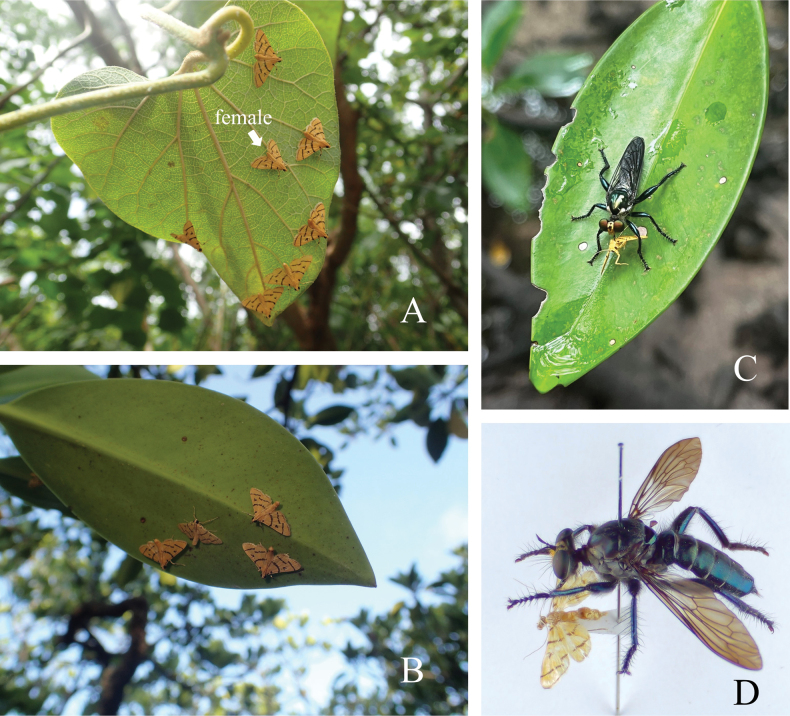
Biology of *Dichocrocisfrenatalis* in Funaura, Iriomote Island, Japan. **A.** Aggregation of adults on the underside of a leaf (23 May 2023), a female is indicated by an arrow, while the others are males; **B.** Aggregation of male adults on the underside of a leaf (26 Aug. 2023); **C.** An adult preyed upon by a mangrove-associated robber fly, *Mairaaenea* (Diptera: Asilidae) [18 June 2024, photo by Nakatada Wachi (@wachinakatada); https://www.inaturalist.org/observations/224300521; accessed on 11 Mar. 2025] **D.** Pinned specimens of the robber fly and its moth prey in Fig. 7C.

Figs 1 , 2 , 3 , 4 , 5 , 6 , 7 (Japanese name: nettai-kurosuji-ki-nomeiga)

Dichocrocis frenatalis Lederer, 1863: 448, pl. 17 fig. 15; Moore 1877: 616; Mally et al. 2019: electronic supplement file 1; Rao and Sivaperuman 2020: 20; Whitaker et al. 2023: 54.
Dichocrocis pandamalis (part): Hampson 1896: 306.

##### Diagnosis.

This species is externally similar to *Conogethespandamalis* but can be distinguished by the continuous postmedial line straight from costa to CuA_2_ in the forewing (in *C.pandamalis* it is interrupted and protruded between M_2_ and CuA_2_), and the narrow and black terminal line in both wings (in *C.pandamalis* the terminal line is lacking). Several undescribed species that are possibly congeneric have been found in Borneo [cf. *Dichocrocis* sp. 1–9 of Whitaker et al. (2023)] and on the Andaman Islands (B. Sumit Kumar Rao pers. comm.), but *D.frenatalis* can be distinguished from these species based on the aforementioned characters. *Dichocrocis* sp. 15 and 16 of Whitaker et al. (2023) most resemble *D.frenatalis*, but they are best distinguished by broad terminal lines on both wings (in *D.frenatalis* they are very narrow).

##### Redescription.

***Head*** (Fig. 1A). Frons yellowish orange, smooth. Vertex with yellow scales, anterior scales porrect, posterior scales erect. Maxillary palpus pale yellow, minute. Labial palpus yellow, upturned, first and second palpomeres with raised scales ventrally. Ocellus distinct, brownish, touching compound eye. Chaetosemata absent. Antennae about 4/5 of forewing length, light brown, dorsally covered with pale yellow scales, ciliate in male, filiform in female; scape yellow. Proboscis covered with yellow scales basally.

***Thorax and legs*.** Thorax yellowish orange dorsally, pale yellow ventrally. Legs almost pale yellow; foreleg tibia with a bold blackish brown band medially; midleg tibia with a pair of spurs, inner spur about 2/3 length of outer one; hindleg tibia with two pairs of spurs, respective inner spurs about 2 times longer than outer ones.

***Wings*** (Fig. 1B–D). Forewing length 6.00–8.80 mm (Suppl. material 1: table S1). The mean forewing length (± SD) was 7.10 ± 0.85 mm for males (*n* = 10) and 7.09 ± 0.71 mm for females (*n* = 10), with no statistically significant difference between sexes (*t* = –0.029, df = 17.387, *p* = 0.978). Forewing ground color yellowish orange; basal fleck black, ending around Cu vein; antemedial line black, gently excurved; postmedial line black, straight from costa to CuA_2_ vein, incurved at a right angle on CuA_2_ vein, then oblique toward dorsum; marginal line black and narrow, running along termen; discocellular lunule black, nearly V-shaped; cell with a small black dot inside the discocellular lunule, but often disappearing; cilia pale yellow, banded with brownish orange medially. Hindwing ground color yellowish orange; discocellular lunule a black bar, often connected with postmedial line; postmedial line black, straight from costa to distal CuP vein, strongly narrowed (often disappearing there) and incurved at an acute angle between veins CuA_2_ and CuP veins, running toward discocellular lunule, then thicken again and extending to dorsum; marginal line as in that of forewing, disappearing around tornus; cilia concolorous with that of forewing. In males, both wings tend to be narrower and each line thicker than in the female. Both wings underneath pale yellowish orange with the same maculation as above, but slightly weaker.

***Wing venation*** (Fig. 2). Forewing Sc and R_1_ separate; R_2_ concurrent with R_3_+R_4_ basally; R_3_ stalked with R_4_ at 1/4 distance from cell; R_5_, M_1_, M_2_, M_3_, CuA_1_, and CuA_2_ separate, almost equidistant; A_1+2_ weakly sinuate near tornus; A_3_ weak, straight; discal cell closed; male retinacular hook absent. Hind wing Sc+R_1_ stalked with Rs at 4/5 of length; M_1_ stalked with Sc+R_1_ at upper angle of cell; M_2_, M_3_, and CuA_1_ close basally; CuA_2_ distant from CuA_1_; CuP and A_1+2_ strong; A_3_ strong, weakly sinuate; female with two frenular bristles.

***Abdomen*.** Yellowish orange; in male, terminal segment enlarged, hair-pencils often visible (Fig. 3A). Tympanal organs (Fig. 3B, C) with praecinctorium not bilobed; tympanum and conjunctivum forming a shallow angle medially; bulla tympani relatively small and narrow, anterior margin truncate; fornix tympani protruded ventrally; saccus tympani extending about 1/3 of 2^nd^ sternite, with straight posterior ridge; venula secundae absent. Male 8^th^ tergite (Fig. 3C) with a broad longitudinal sclerotization, its anterior end Y-shape, lateral margins tapered posteriorly, posterior end with strong wrinkles; both sides of posterior portion with a short linear sclerotization. Male 8^th^ sternite with two longitudinal sclerotizations, connected to strongly sclerotized and anteriorly convex anterior margin (Fig. 3C).

***Male genitalia*** (Figs 4, 5). Tuba analis (ta) weakly sclerotized, about 2.5 times longer than uncus. Uncus narrow, curved ventrad, apex covered with ventrally directed and bifid chaetae. Tegumen somewhat broad, tapered toward uncus. Parateguminal sclerite [*sensu*Solis et al. (2020)] extending from tegumen complicated in structure, composed of a saddle-shaped pad (sp) dorsally and a larger triangular sclerite (ts) ventrally, each with hair-pencils consisting of six types of specialized scales: the entire sclerite with many tufts of slender spatulate scales (ss), the dorsal and ventral tufts strongly bent inwardly, otherwise bent in various directions; the saddle-shaped pad dorsally with a tuft of somewhat broad spatulate scales (bs) that ventrally bent and distally tapered; the triangular sclerite with a tuft of long, lamellate scales (ls) at its apex, and a cluster consisting of short, blackish brown filiform scales (fs1), longer and brown filiform scales (fs2), and short hock-shaped scales (hs) internally. Gnathos absent. Saccus (sa) large and broad in dorsoventral view, rounded. Valva costa with a straight sclerotized ridge (sr) extending distal 1/4 and a large rounded basal bulge (bb), costal margin strongly concave at the termination of the ridge, dorsally with dense hair-like setae; inner surface with a wavy furrow (wf) running from apex to basal 2/3; ventral margin subtriangularly bulged medially, its apex with a tuft of long hair-like setae (vs); fibula (fb) spatula-shaped, basally separates into two arms, ventral arm concurrent with saccular base; sacculus (sl) a membranous band. Transtilla narrow, weakly connected medially. Juxta (jx) elongate, medially constricted with two longitudinal ridges, dorsal margin truncate, ventral margin medially concave and laterally connected to basal sacculus. Phallus (ph) with antero-ventrally extended caecum; vesica covered with fine microspines, without cornuti.

***Female genitalia*** (Fig. 6). Papillae anales broad. Anterior apophyses slightly curved, apex blunt. Posterior apophyses broader than anterior ones, dilated near base. Antrum somewhat narrow, membranous, funnel-shaped. Ductus bursae membranous, sclerotized near corpus bursae, lateral margins of the sclerotization lightly tapered (Fig. 6C). Ductus seminalis emerging from the posterior end of ductus bursae. Spermathecal gland with lagena. Corpus bursae ellipsoid, 1/5 from anterior end with a transverse band composed of needle-like spines (ca 0.15–0.30 mm, Fig. 6D), and anterior portion from it sparsely covered with similar but shorter spines (ca 0.07–0.09 mm, Fig. 6D).

##### Material examined (n = 212 individuals).

**Okinawa I.** • 2♂; Nago-shi, Ôura; 1 July 2009; U. Jinbo; NSMT. **Ishigaki I.** • 1♀; Takeda-rindô; 21 May 2009; U. Jinbo; NSMT; • 1♂; Yarabe-rindô; 22 Aug. 2022; I. Aoki; gen. slide no. YM878; ELKU. **Iriomote I.** • 1♀; Tedô-san parking; 30 May 2015; Y. Matsui; LT; ELKU; • 1ex.; ditto; 5 Apr. 2024; T. Mano; LT; ELKU; • 1♂; Sonai; 11 Jan. 2023; Y. Yoshiyasu; OMU; • 1♂1♀; ditto; 4 June 2024; T. Mano; • 2♀; Haemi; 22 to 24 Nov. 2019; F. Ishiwata; gen. slide no. YM876, 877; ELKU; • 3♀; ditto; 6 Oct. 2024; T. Mano; • 4♂5♀; Komi; 25 May 2009; U. Jinbo; NSMT; • 1♂; Funaura; 22 Sep. 2000; M. Kinjo & S. Katada; RUMF; • 1♀; ditto; 4 Oct. 2000; M. Kinjo & S. Katada; RUMF; • 1♂; ditto; 18 Jan. 2001; M. Kinjo & S. Katada; RUMF; • 1♀; ditto; 25 May 2009; U. Jinbo; NSMT; • 3♂2♀; ditto; 4 Apr. 2023; N. Wachi; TBRC and ELKU; • 5♂3♀; ditto; 21 Apr. 2023; N. Wachi; TBRC and ELKU; • 2♂; ditto; 24 Apr. 2023; N. Wachi; TBRC; • 4♀; ditto; 2 May 2023; N. Wachi; TBRC; • 3♀; ditto; 5 May 2023; N. Wachi; TBRC; • 5♂2♀; ditto; 20 May 2023; N. Wachi; TBRC; • 7♂1♀; ditto; 23 May 2023; N. Wachi; TBRC and ELKU; • 4♂1♀; ditto; 3 June 2023; N. Wachi; TBRC; • 5♂2♀; ditto; 6 June 2023; N. Wachi; TBRC and ELKU; • 4♀; ditto; 26 June 2023; N. Wachi; TBRC; • 4♂6♀; ditto; 4 July 2023; N. Wachi; TBRC; • 2♂2♀; ditto; 7 July 2023; N. Wachi; TBRC; • 2♂4♀; ditto; 12 July 2023; N. Wachi; TBRC; • 4♂4♀; ditto; 14 July 2023; N. Wachi; TBRC; • 1♂1♀; ditto; 13 Mar 2024; N. Wachi; TBRC; • 1 ex.; ditto; 4 Jun 2024; N. Wachi; prey of *Mairaaenea* (Fabricius) (Diptera: Asilidae); iNaturalist observation ID: 220722951; TBRC; • 1 ex.; ditto; 18 Jun 2024; N. Wachi; prey of *M.aenea*; iNaturalist observation ID: 224300521; TBRC; • 2 ♂; ditto; 26 Mar 2025; N. Wachi; TBRC; • 4♂3♀; ditto; 9 Apr 2025; N. Wachi; TBRC; • 1♀; ditto; 3 Dec. 2024; Y. Matsui & N. Wachi; ELKU; • other 90 individuals; ditto; N. Wachi; TBRC (see Suppl. material 2: table S2).

##### Distribution.

India (Nicobar Islands) (Lederer 1863), Indonesia (Sumatra Island) (Mally et al. 2019), Hong Kong (Whitaker et al. 2023), Japan (Okinawa, Ishigaki, and Iriomote Islands) (this study).

##### Biological notes.

Our collection data suggest that the adults occur almost year-round. In the mangrove environment of Funaura, Iriomote Island, aggregations of adults on the underside of leaves were occasionally observed during the day (Fig. 7A, B). The aggregations included both sexes, although males were significantly more numerous. Some adults were observed being preyed upon by a robber fly, *Mairaaenea* (Fabricius) (Diptera, Asilidae) (Fig. 7C, D). The occurrence of this robber fly in mangrove environments has been reported in previous studies (Utsugi 2006; Tomazovic and Grootaert 2010).

##### Tribal placement.

The results from our molecular phylogenetic analysis placed *D.frenatalis* in Steniini (see below). In addition, the following genital characters, as proposed by Mally et al. (2019) for Steniini, support this placement: bifid uncus chaetae, undulated valva costa, well-separated valva fibula and distal sacculus, broad saccus, phallus with caecum, and corpus bursae with spinose texture and lacking signa. Since *D.frenatalis* is the type species of *Dichocrocis*, we assign the genus to the tribe Steniini.

##### Remarks.

In this study, we identified the Japanese specimens of this species as *D.frenatalis* based on the wing maculation of the type specimen (abdomen missing) preserved in the Natural History Museum, London, UK (NHMUK) (Fig. 1B), in addition to the original description. We consider this identification to be justified at this time for the following reasons: 1) the abdomen of the type specimen is lost, rendering identification based on genitalia impossible, 2) no additional specimens of this species have been collected at the type locality (B. Sumit Kumar Rao pers. comm.), and 3) although Whitaker et al. (2023) illustrated many (putative) *Dichocrocis* species, none of them exhibit wing maculation matching that of *D.frenatalis*.

### ﻿Molecular phylogenetic analysis

**Figure 8.**
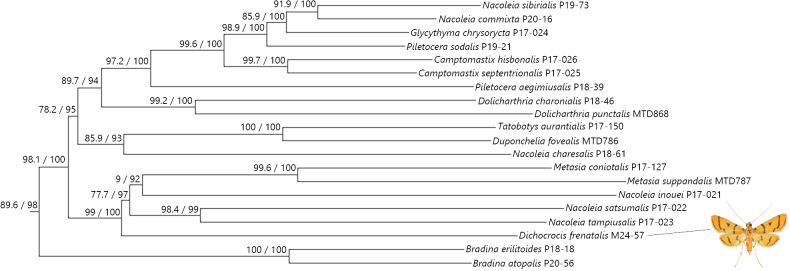
A maximum-likelihood (ML) phylogeny based on four-gene dataset (the parts other than the tribe Steniini are omitted). The numbers at each node indicate the Shimodaira-Hasegawa-like approximate likelihood ratio tests (SH-aLRT) / ultrafirst bootstrap (UFBT).

The entire COI gene, partial CAD, RpS5, and EF1α genes from the Japanese specimen of *D.frenatalis* were sequenced for the first time. No matching or closely related sequences were found in the BOLD (https://boldsystems.org/) or GenBank (https://www.ncbi.nlm.nih.gov/genbank/) databases. *Dichocrocisfrenatalis* is recovered within the strongly supported monophyletic Steniini (SH-aLRT = 89.6, UFBT = 98) in the maximum likelihood tree (Fig. 8). Most relationships within Steniini are significantly supported (SH-aLRT ≥ 80, UFBT ≥ 95), and *D.frenatalis* is the sister taxon to a clade consisting of *Metasia* and paraphyletic *Nacoleia* [“group 1” *sensu*Matsui et al. (2022)] species with near-maximum support values (SH-aLRT = 99, UFBT = 100).

## ﻿Discussion

In this study, we provide the first detailed redescription of *Dichocrocisfrenatalis*, the type species of *Dichocrocis*. The genus is taxonomically problematic as a polyphyletic and speciose genus within Spilomelinae (Mally et al. 2019). This issue likely originates from Hampson’s (1896) taxonomic treatment, which synonymized *D.frenatalis* with *C.pandamalis*. These two species belong to distant lineages: the former is in the Steniini, as shown in this study, while the latter belongs to the genus *Conogethes* in the tribe Margaroniini. Since *C.pandamalis*, erroneously regarded by Hampson (1896) as the type species of *Dichocrocis*, is a member of Margaroniini, several species of Margaroniini are still misplaced in *Dichocrocis*. For example, molecular and morphological analyses by Mally et al. (2019) confirmed that *D.zebralis* (Moore, 1867) belongs to the *Glyphodes* genus group sensu Sutrisno (2002) in Margaroniini. In addition, the male and female genitalia of *D.klotsi* Pajni & Rose, 1977, as described in the original description, suggest that its placement near or in *Conogethes*. The morphological information of the type species provided here should serve as a basis for re-assessing the placement of other members currently assigned to *Dichocrocis*.

The males of *D.frenatalis* have remarkably well-developed, complex hair-pencils compared to other Spilomelinae species. Similarly complicated hair-pencils are also found in *Camptomastix* and *Piletocera* species in the same tribe (Mally et al. 2019; YM pers. obs.). Generally, hair-pencils attached to male genitalia are believed to be associated with pheromone release and/or storage (Birch et al. 1990), although their function has been experimentally demonstrated in only a few studies, including Spilomelinae. One such example is *Conogethespunctiferalis*, in which the male has hair-pencils consisting of four types of scales, and washing these scales with an organic solvent or removing them significantly reduces their mating success (Kimura and Honda 1999; Kimura et al. 2002). Interestingly, the four types of male hair-pencil scales in *C.punctiferalis* exhibit functional differentiation: male pheromones are contained exclusively in the retiform and filiform scales, which are located inside the hair-pencil-complex and are easily detached, whereas the outer phylliform and spatulate scales appear to protect the inner scales (Kimura et al. 2002). The six types of scales found in *D.frenatalis* likely exhibit functional differentiation, similar to those in *C.punctiferalis*: the filiform scales are located inside the hair-pencil complex and are easily detached, while the other scales are relatively robust and cover the filiform scales. Further investigation using electron microscopy and chemical analyses will help elucidate the function of the hair-pencil scales in *D.frenatalis*.

Given that the adults of *D.frenatalis* were found in mangrove environments in this study, their life history is probably closely associated with mangroves, although the immature stages of this species remain unknown. Most species of Steniini are detritivorous as far as known (Mally et al. 2019; Matsui et al. 2022). However, while the detritivorous species in this tribe easily lay eggs on the walls of housed plastic containers, the tested females of *D.frenatalis* did not do so (YM pers. obs.), suggesting a different feeding habit. Two other members of Steniini, *Tatobotysjanapalis* (Walker) and *T.aurantialis* Hampson are also abundant in mangrove habitats. The former feeds on mangrove flowers and buds (Murphy 1990), and the latter on red algae submerged in seawater (Yoshiyasu 2017). As with these species, *D.frenatalis* may depend on specific resources other than detrital matter in mangrove environments.

Daytime aggregations of adults on the underside of leaves have been reported in some hygrophilous crambid taxa, including *Eristena* and *Strepsinoma* (Acentropinae) (Yoshiyasu 1984; Robinson et al. 1994; Speidel and Mey 1999), as well as *Taurometopa* (Odontiinae) (Murphy 1990), but, to our knowledge, not in Spilomelinae. Murphy (1990) considered the assemblage of *T.pyrometalla* Meyrick as a “lek” because all members of the assemblage were males with sexually dimorphic (larger) heads. The observed assemblages of *D.frenatalis* include more males than females, but do not exhibit visible sexually dimorphic characters (except for the structure of abdominal tip). In the case of *D.frenatalis*, it is more likely that the males are attracted to pheromone-releasing females on the underside of leaves at night, and remain there during the day. Further investigation of the mating behavior of each species will help clarify the behavioral factors underlying the formation of crambid adult assemblages.

The known localities of *D.frenatalis* are discontinuous: the Nicobar Islands, Sumatra, Hong Kong, and the Ryukyu Islands. Moreover, this species has not been rediscovered at its type locality, the Nicobar Islands, in recent years (B. Sumit Kumar Rao pers. comm.). In this study we found that *D.frenatalis* is abundant in mangrove environments. The scarcity of records for this species may reflect its specialized habitat. Further surveys of mangrove environments may lead to its rediscovery in known localities or discovery of new habitats in other regions. However, *D.frenatalis* was not detected in Murphy’s (1990) comprehensive survey of Singapore mangrove environments, which reported 102 herbivorous insect species attacking nine principal tree taxa. This suggests that *D.frenatalis* is likely absent from Singapore mangrove environments, and therefore it cannot be ruled out that the possibility that the distribution of this species is highly localized.

## Supplementary Material

XML Treatment for Dichocrocis frenatalis

## ﻿Additional information

### Author ORCIDs

Yuki Matsui https://orcid.org/0000-0002-0434-7111

Nakatada Wachi https://orcid.org/0000-0001-9958-5093

### Data availability

All of the data that support the findings of this study are available in the main text or Supplementary Information.

## Supplementary materials

Supplementary material 1 Forewing lengths (mm) of male and female *Dichocrocisfrenatalis* specimens used in the statistical analysis Data type xlsx Explanation note **table S1**: Forewing lengths (mm) of male and female *Dichocrocisfrenatalis* specimens used in the statistical analysis. M = male; F = female. “#” indicates the sample number corresponding to Suppl. material 2: table S2. R script used for statistical analysis of forewing length data. A Welch’s t-test was conducted using R to compare the mean forewing length between male and female specimens. https://binary.pensoft.net/file/1361041This dataset is made available under the Open Database License (http://opendatacommons.org/licenses/odbl/1.0/). The Open Database License (ODbL) is a license agreement intended to allow users to freely share, modify, and use this Dataset while maintaining this same freedom for others, provided that the original source and author(s) are credited.Yuki Matsui, Nakatada Wachi, Yutaka Yoshiyasu

Supplementary material 2 Detailed information for *Dichocrocisfrenatalis* specimens examined in this study Data type xlsx Explanation note **table S2**: Detailed information for *Dichocrocisfrenatalis* specimens examined in this study. Sample number (“#”), collection start and end days, month, year, total number of individuals (N Ind), number of males (N male), number of females (N female), locality island, detailed locality, preservation and preparation status of specimens (Specimen), collector (Collected by), associated species or ecological context (Associated), and additional notes (Note). Some sample numbers correspond to those shown in Suppl. material 1: table S1. https://binary.pensoft.net/file/1361042This dataset is made available under the Open Database License (http://opendatacommons.org/licenses/odbl/1.0/). The Open Database License (ODbL) is a license agreement intended to allow users to freely share, modify, and use this Dataset while maintaining this same freedom for others, provided that the original source and author(s) are credited.Yuki Matsui, Nakatada Wachi, Yutaka Yoshiyasu
